# Toxicity, Chemistry, and Public Health Relevance of Emerging Nicotine Analog Vapes, Pods, and Pouches

**DOI:** 10.1021/acs.chemrestox.5c00537

**Published:** 2026-03-31

**Authors:** Rhea Raghu, Mohana Sengupta, Karen Lin, Felix Effah, Robert M. Strongin, Irfan Rahman

**Affiliations:** † Department of Environmental Medicine, 6923 University of Rochester Medical Center, Rochester, New York 14624, United States; ‡ Department of Chemistry, 6685 Portland State University, Portland, Oregon 97201, United States

## Abstract

Electronic nicotine delivery system manufacturers, such as Charlie’s Holdings Inc., ECBlend, Outlaw, and NicRiver, have recently introduced nicotine analogs, such as 6-methyl nicotine, 6-MN (“Metatine”), and nicotinamide, NA (“Nixamide,” “Nixodine,” or “Nixotin-free base and salt”) in products to circumvent the U.S. FDA’s premarket tobacco product application (PMTA) requirements. Marketed as “tobacco-free,” “PMTA-exempt,” or “FDA-approved,” these compounds now appear in oral nicotine pouches and disposable bars/vapes from brands such as Outlaw Dip, Kumi-Six, SBX, Katchmi, and Spree Bar under proprietary labels including “NoNic6,” “Metatine,” or “NIC-SAFE.” These products often mimic the appeal of conventional nicotine delivery systems, with extensive use of fruit, menthol, and candy-inspired flavorings. Independent testing, however, has revealed inconsistencies between labeled and actual concentrations of 6-MN, alongside the presence of undisclosed chemical additives such as “coolants” and numerous other examples. Additionally, emerging toxicological evidence indicates that 6-MN is more potent than nicotine to cause oxidative, inflammatory, and toxic responses. This includes the activation of NF−κΒ, causes epithelial permeability, and lung remodeling due to extracellular matrix (ECM) modifications. Misleading claims by industry sources include erroneous statements that imply nicotinamide interacts with nicotinic acetylcholine receptors (e.g., methylated nicotine analogs-interaction with α4β2 nicotinic acetylcholine receptors -nAChR interactions). Despite health risks, regulatory frameworks remain ambiguous, enabling companies to circumvent oversight by exploiting loopholes around synthetic analogs. There is a need for rigorous chemical and toxicological studies to evaluate the health effects of nicotine analogs, such as 6-MN and NA, and their interactions with flavorings. This review summarizes current knowledge of chemistry, pharmacology, toxicity, product landscape, flavoring profiles, and labeling practices of 6-MN- and nicotinamide-containing and nicotinamide products, highlighting the urgent need for regulatory clarity, transparent labeling, and further chemico-toxicological assessment.

## Graphical abstract

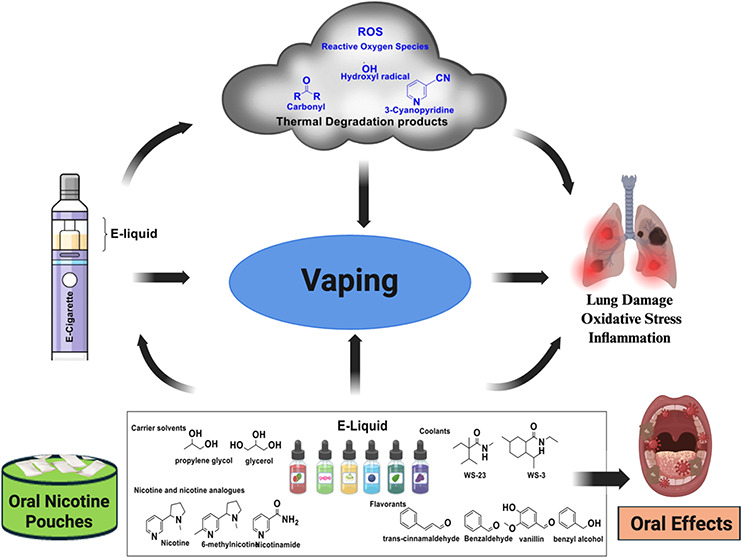

## Introduction

Electronic nicotine delivery systems (ENDS) have diversified beyond tobacco-derived nicotine, with manufacturers including Charlie’s Holdings Inc., ECBlend, Outlaw, Aroma King, NicRiver, and Mi-One introducing synthetic analogs to evade the FDA’s stringent PMTA process. These emerging ENDS products featuring novel menthol and tobacco-flavored nicotine analogs, such as 6-methyl nicotine (6-MN) in Spree Bar and nicotinamide (NA) in EC Blend and Outlaw Dip, are also growing in popularity among users, including adolescents and young adults. This is due to marketing to attract youth with artificial intelligence-generated characters, artificial fruity flavorings, and unique devices such as dip pouches, oral nicotine pouches, and disposable bars sold by various tobacco vendors. Pouches often consist of nicotine and nontobacco components such as artificial additives, flavorings, and sweeteners, appealing to young adults.^1^ Other additives such as synthetic cooling agents with analgesic effects are also often encountered in these products that are not well studied yet.^1^ Tobacco smoking and nicotine use, especially with the proliferation of synthetic nicotine analogs, such as 6-MN and NA can present significant public health risks by introducing harmful reactive chemicals into the lungs, contributing to respiratory disorders.

Tobacco products containing nicotine are currently regulated by the Food and Drug Administration (FDA)^2^ under the U.S Tobacco Control Act (TCA) by requiring tobacco manufacturers to submit a premarket tobacco product application (PMTA) before legal marketing.^3^ However, nicotine analogs and other non-nicotine tobacco alkaloids can be utilized by companies to circumvent federal schemes focusing on nicotine alone,^4^ creating regulatory loopholes and ambiguities.

The rapid proliferation of 6-MN and NA products underscores a gap between chemical innovation and regulatory preparedness. Recent studies have used analytical chemistry techniques such as gas chromatography and mass spectrometry (GC/MS) and liquid chromatography with high resolution (HRLC/MS/MS) to quantify 6-MN and other methyl nicotine analogs in nicotine products such as leaf and smokeless tobacco. In these studies, researchers confirmed the natural presence of 6-MN to average around 0.32 μg/g across all samples.^4^ Products containing 6-MN had also been found to contain nicotine contents ranging from 8 to 25 mg per oral pouch, higher than traditional pouches,^5^ in turn implying an increased potential toxicity compared to conventional nicotine. However, because most commercial nicotine is extracted and purified from tobacco, 6-MN can be a minor alkaloid in most products.^4^

Furthermore, despite being promoted as safer alternatives, recent research found 6-MN to exhibit greater toxicity than traditional nicotine in human bronchial epithelial cells.^6^ In both submerged cultured systems and air–liquid interface (ALI) exposure models, 6-MN produces higher levels of reactive oxygen species (ROS) and enhanced cytotoxicity relative to nicotine, posing great risks to pulmonary diseases.^6^ In other in vitro studies of the effect of 6-MN on cytotoxicity and gene expression effects compared to BEAS-2B human bronchial epithelial cells via assays, researchers concluded that BEAS-2B exhibited a greater sensitivity to 6-MN compared to nicotine^7^ by activation of NF-κB, MET, and EGFR compared to traditional nicotine.^7^ This greater oxidant generation is driven in part by heavy metal leaching from high-resistance heating coils.^8^

One potential mechanism underlying metal-associated ROS generation, as described in Effah et. al (2025), is Fenton-like redox chemistry, where transition metals like iron or copper catalyze the conversion of hydrogen peroxide into highly reactive hydroxyl radicals.^8^ However, as metals may also enhance oxidative stress through alternative mechanisms, this is unlikely to be the sole pathway. Other avenues include disruption of cellular redox signaling, mitochondrial dysfunction, and induction of inflammatory responses.^8^ Together, these findings highlight that both chemical formulation and aerosol generation contribute to this observed pulmonary toxicity.

Additionally, if further regulatory oversight and strict regulations are not implemented on 6-MN and NA containing products, new vendors can take advantage of the “PMTA-exemption”, further contributing to their commercial spread of 6-MN and NA products, attracting youth. Although nationally representative prevalence data specific to 6-MN and NA remains limited, market surveillance indicates rapid commercialization and consumer availability of nicotine analog products in the US and internationally. Public health surveillance data suggest that awareness and use of these nicotine analogs are already significant among young people. For example, a recent survey of adolescents oversampled for prior tobacco use found that approximately 20% were aware of products containing these analogs, and 8% had used them within 6 months of their introduction to the market.^9^ Ultimately, the limited population-level data should be interpreted as an emerging surveillance gap rather than lack of evidence of risk for these analogs.

Still, the toxicity and cytotoxicity of 6-MN are not well studied to date. This review consolidates the current understanding of 6-MN and NA chemical reactivity, aerosol profiles, and biological activity. Additionally, a comprehensive comparison of flavorings, labeling, and product marketing practices among current commercially available 6-MN and nicotine analog products (Outlaw Dip, Kumi, Katchmi, SBX, and Spreebar) is described as well as public health implications.

## Chemical Structure and Thermal Degradation of 6-Methyl Nicotine and Nicotinamide

Nicotine, 6-MN and NA each contain a pyridine ring as a defining structural motif, but subtle substitutions dramatically alter pharmacology and chemical behavior.^8^ Traditional nicotine consists of (*R)* and (*S)-*enantiomers, where (*S*)-nicotine is typically more potent than (*R*)-nicotine^10,11^ (Table 1). In contrast, 6-MN differs from conventional nicotine by the presence of a methyl group at the 6-position of the pyridine ring^2,5^ (Table 1). This addition of a methyl group increases hydrophobicity, modifies the molecule’s dipole moment, and enhances binding affinity to α4β2 nicotinic acetylcholine receptors (nAChRs),^12,13^ consequently producing similar sensations and possibly conferring greater addiction potential than nicotine.

**Table 1:**
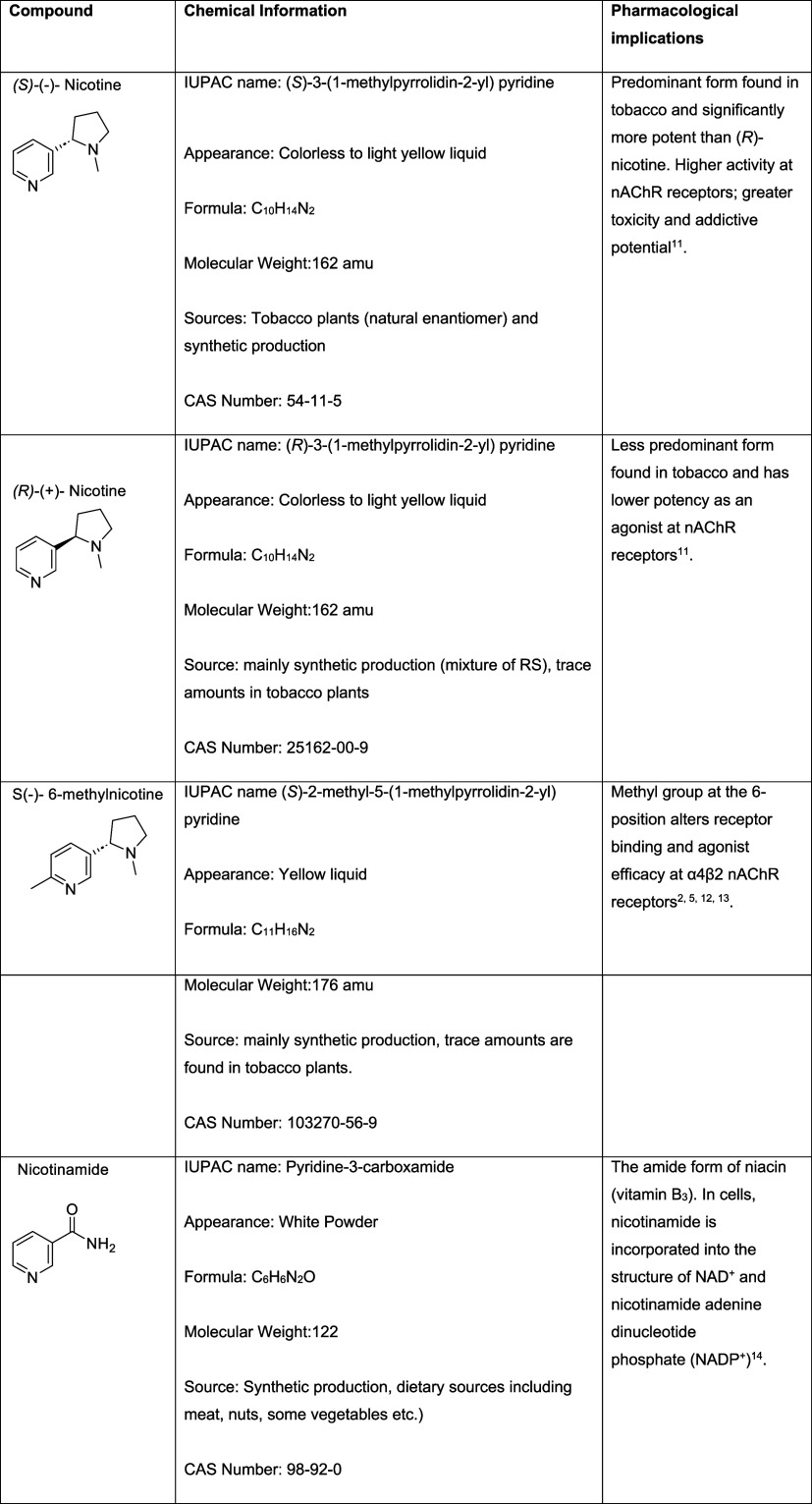
Molecular Formulas, Pharmacological Implications, and Chemical Structures of Nicotine, 6-Methyl Nicotine and Nicotinamide

Nicotinamide (NA), conversely, possesses a pyridine ring but contains an amide group instead of a pyrrolidine moiety (Table 1). This will alter chemical degradation pathways during vaping and will impact biological properties. Additionally, NA is the amide form of niacin (vitamin B_3_). In cells, nicotinamide is incorporated into the structure of NAD^+^ and nicotinamide adenine dinucleotide phosphate (NADP^+^).^14^

## Thermal Degradation Pathways of Nicotine, 6-MN and NA

(*S*)-Nicotine has been extensively studied due to its long association with conventional cigarette smoking, wherein combustion temperatures exceed 700 °C.^15^ At these high temperatures, nicotine undergoes pyrolytic unimolecular dissociation involving significant bond cleavage with high activation energies, producing well-characterized thermal degradation products during smoking such as myosmine, nornicotine, 3-ethynylpyridine, and 3-cyanopyridine^16^ (Figure 1). Many other degradation products are also formed during cigarette smoking at such high temperatures.^15^

**Figure 1:**
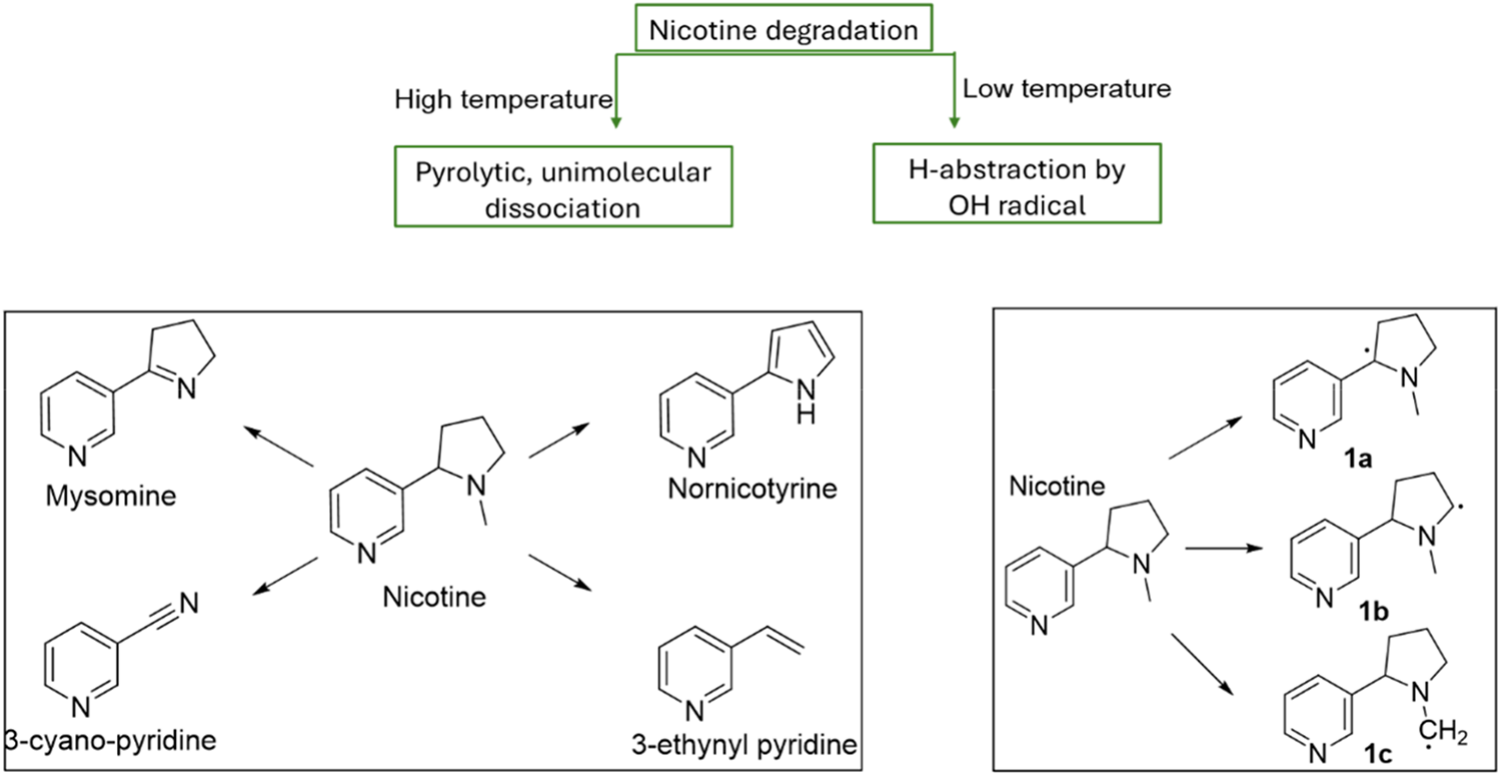
Different mechanistic pathways of nicotine thermal degradation at high (cigarette) and low (e.g., e-cigarette) temperatures. Left: pyrolysis products. Right: Hydroxyl radical hydrogen-abstraction intermediates Figure 1a–c. The left figure is adapted from Asensio et al.^16^ The right figure is adapted from Hoa et al.^17^

In contrast to conventional cigarettes, e-cigarettes operate at lower temperatures (typically ∼ 200–400 °C), wherein nicotine degradation will follow a different mechanistic pathway dominated by hydrogen abstraction via hydroxyl radicals.^15^ This mechanism exhibits non-Arrhenius kinetics, with reactivity increasing as temperature decreases. Although e-cigarettes are often perceived as less harmful due to their noncombustive, lower temperature operation, these alternative degradation pathways will also generate reactive and toxic intermediates, challenging the assumption of greatly reduced risk.^15^

## Secondary Reactions and Radical-Mediated Chemical Pathways in E-Cigarettes

Hydrogen abstraction by hydroxyl radicals is a key radical-mediated degradation pathway that generates the reactive radical intermediate species noted in Figure 1 (1a–1c).^17^ These radicals subsequently form various carbonyls and related toxicant compounds via reacting readily with O_2_ and e-liquid constituents. These reactions produce complex e-cigarette emission mixtures of toxic degradation products, including aldehydes, ketones, and substituted pyridine derivatives.^18,19^

## Potential Thermal Degradation Pathway of 6-Methylnicotine (6-MN)

6-MN shares structural similarity with nicotine but contains an additional methyl group on the pyridine ring, which will alter its reactivity and thermal degradation behavior compared to nicotine (Figure 2). While the following degradation pathways are chemically plausible for 6-MN, direct confirmation under ENDS conditions remains limited, and these mechanisms should be interpreted as potential rather than established processes.

**Figure 2:**
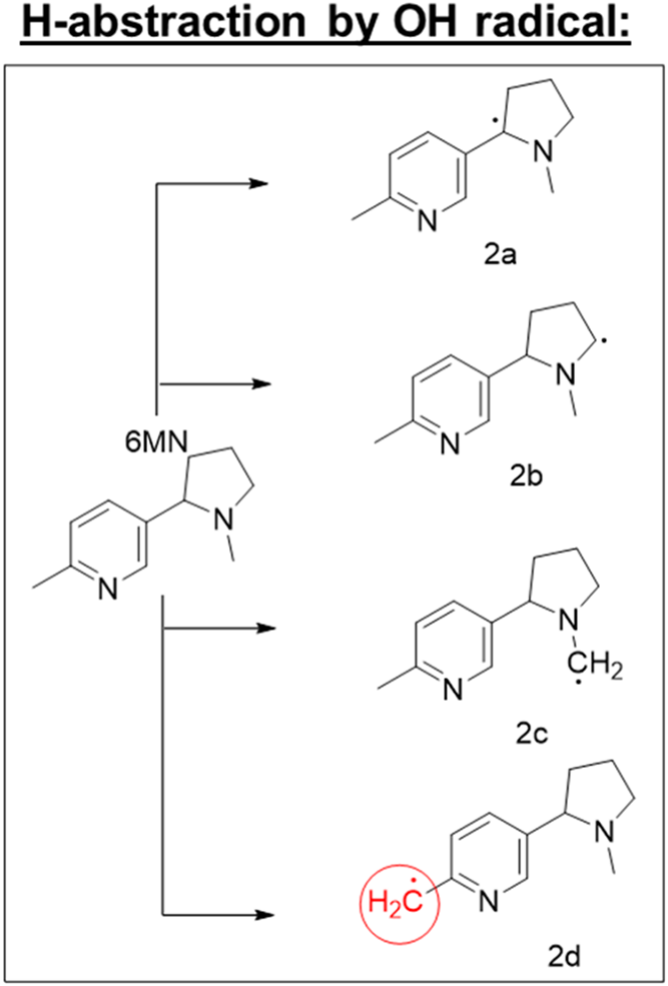
Proposed radical intermediates from the preferred hydrogen atom abstraction sites of 6-MN at vaping-relevant temperatures.

Under (low-temperature) vaping conditions, 6-MN is expected to undergo hydrogen abstraction by hydroxyl radicals in a manner similar to nicotine, as shown in Figure 1. However, the 6-methyl group introduces an additional reactive site, enabling alternative radical formation pathways and distinct aerosol products (Figure 2). Radical (Figure 2d) is potentially significant due to its resonance stabilization via the conjugated aromatic ring. Radical (Figure 2d) would serve as an additional radical interacting with other reactive e-liquid components, generating complex mixtures of products with currently unknown toxicological implications.

## Thermal Degradation Pathways of Nicotinamide

Under e-cigarette operating temperatures, NA can potentially thermally degrade to yield several byproducts, including pyridine derivatives, ammonia, nitroso compounds, and *N*-oxides.^2^ It has recently been reported that, under e-cigarette usage condition, NA undergoes a reversible dehydration mechanism to form a major product, 3-cyanopyridine (3-CP, Figure 3).^20^ This result is consistent with the fact that 3-CP is a well-known, major industrial byproduct of nicotinamide (NA), generated via heating and dehydration of NA at temperatures in range of those encountered during e-cigarette operation.^21,22^ In contrast, the formation of 3CP from nicotine (Figure 1) requires relatively higher temperatures. Under heating and aerosolization conditions, the formation of 3CP from NA was observed to increase sharply at temperatures between 250 and 335 °C (Figure 3).^20^ Analysis of vaped Nixamide e-liquid (Nicotine River) confirmed that nicotinamide undergoes thermal degradation under degradation under realistic vaping conditions.^20^ The chemical profile of the resulting aerosol mirrored laboratory-models, specifically identifying 3-cyanopyridine as the primary degradant. This alignment suggests that laboratory simulations accurately predict the chemical transformations occurring within commercial “nicotine-alternative” devices.^20^ Pyridine can also be generated from either NA or 3CP, but this typically occurs at higher temperatures; for instance, pyridine formation has been observed at 675 °C, whereas only trace amounts are detected at 300 °C.^20^ These observations highlight how structural differences between nicotine analogs and nicotine dictate their thermal degradation profiles and the nature of the toxicants produced under vaping-relevant conditions.

**Figure 3:**
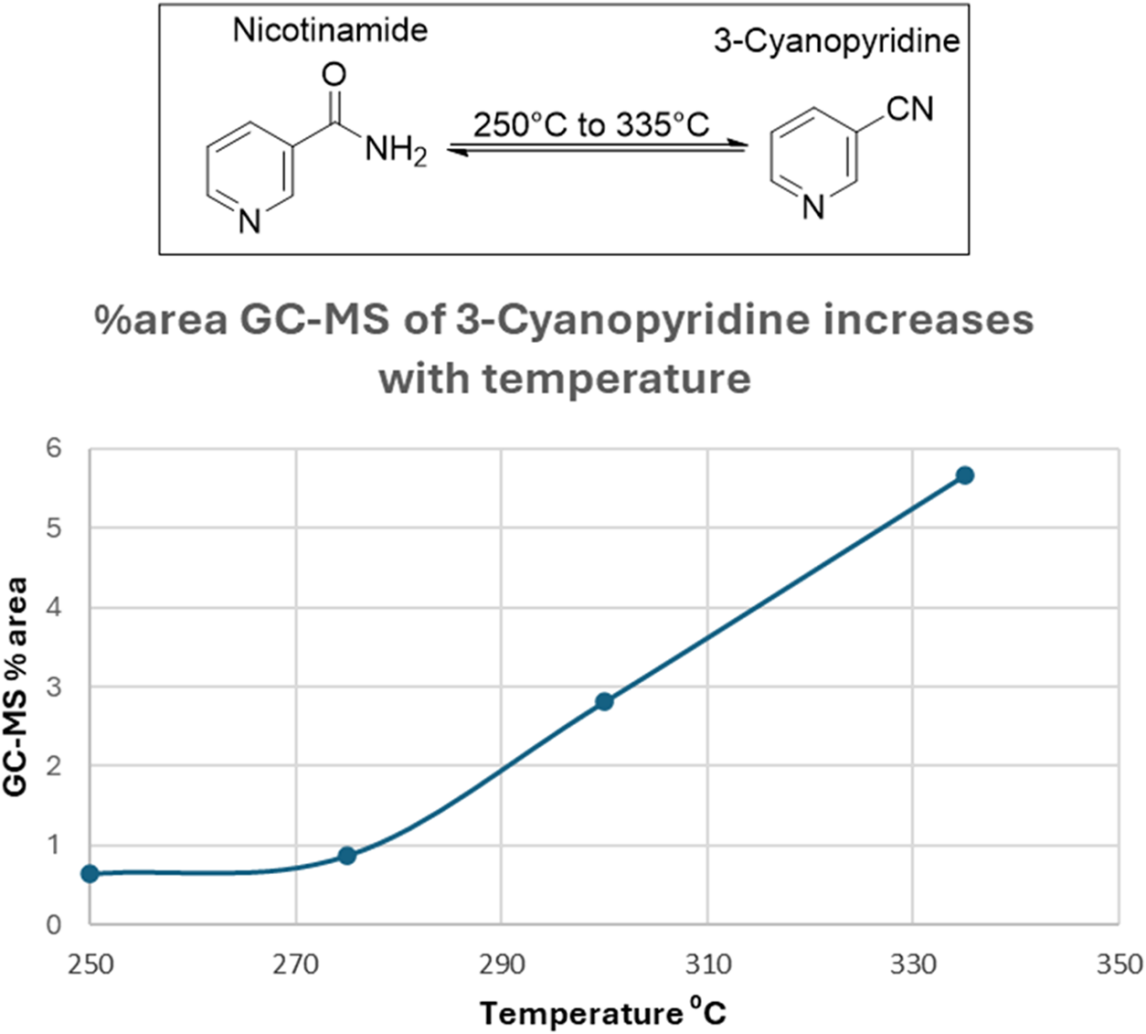
Temperature-dependent formation of 3-CP from nicotinamide. The 3-CP yield increases with temperature, showing progressive growth from 250 to 335 °C. Reprinted with permission from ref 20. Copyright 2025 by the American Chemical Society.

The thermal degradation of NA and its byproducts also suggest a possibility of generating toxic volatile species such as hydrogen cyanide (HCN) and ammonia (NH_3_).^23^ Mechanistically, the conversion of 3-CP to pyridine can release HCN, while further breakdown of pyridine in aqueous media has been reported to produce NH_3_.^23^ Both species are highly toxic to humans, raising health concerns for individuals exposed to vaporized products containing these precursors.

Biological evidence further supports the toxicological relevance of 3-CP. Even trace concentrations of 3-CP (0.0001 ppm) have been discovered to exert cytotoxic effects in human lung epithelial BEAS-2B cells.^20^ Notably, coexposure to nicotinamide and low levels of 3-CP (0.000001 ppm) results in a synergistic interaction that significantly enhances overall cytotoxicity beyond the effect of either compound alone (Figure 4).^20^ This finding shows that even if NA may generate relatively low levels of 3-CP under vaping-relevant temperatures, the concurrent presence of both compounds can amplify toxicity.^20^ These results emphasize that individual e-liquid constituents may appear minimally harmful in isolation, yet their thermally generated mixtures can exhibit enhanced or emergent toxicological properties.^18^

**Figure 4:**
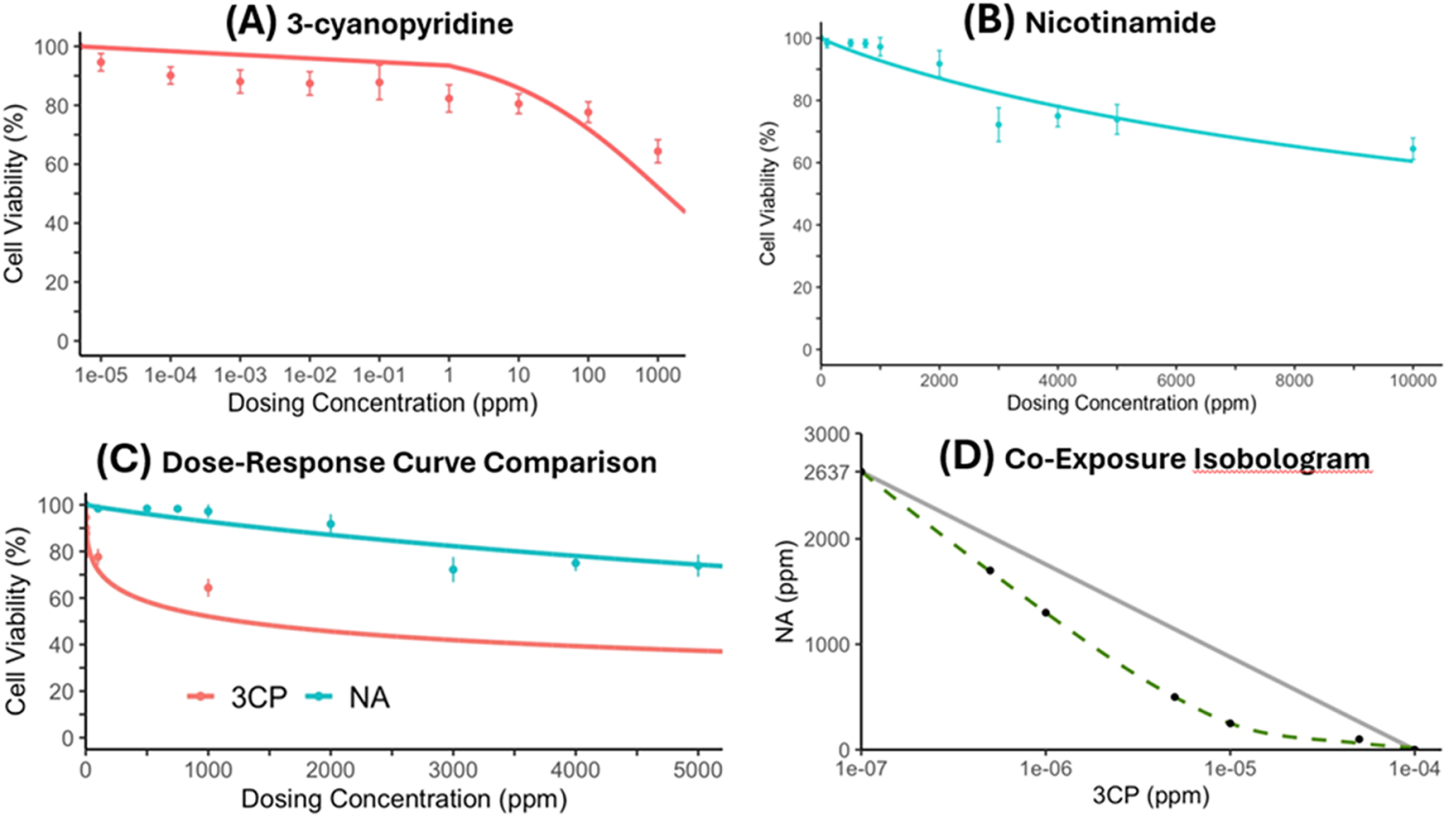
LC_10_ dose response (DR) curves and chemical interaction model of 3-cyanopyridine (3CP) and nicotinamide (NA) tested on BEAS-2B cells. (A) DR of 3CP graphed on the log-transformed *x*-axis of concentrations tested up to 1000 ppm. (B) DR of NA tested at concentrations up to 10,000 ppm. (C) The combined DR curves of 3CP and NA are plotted on the same concentration scale. The dose–response curves are identical to those shown in panels A and C, but on a comparable scale. The 3CP curve above 1000 ppm is a model prediction based on the curve produced at lower concentrations, rather than on tested values. (D) Isobologram of 3CP:NA, indicating synergistic interaction. Reprinted with permission from ref 20. Copyright 2025 by the American Chemical Society.

Beyond its direct cytotoxicity, 3-CP exhibits remarkable coordination versatility, functioning as either a terminal or bridging ligand with transition metals. Structural investigations have shown that complexes of the type [M^2+^Br_2_(3-CP)_4_] (M = Mn, Fe, Co, Ni) form discrete octahedral geometries that, upon heating, evolve into polymeric [M^2+^Br_2_(3-CP)_2_]*_n_* and [M^2+^Br_2_(3-CP)_1_]*_n_* frameworks in which 3-CP bridges adjacent metal centers through both pyridine and cyano nitrogen atoms.^24^ These findings clearly demonstrate the capacity of 3-cyanopyridine to coordinate and bridge divalent 3d metal ions. The coordination chemistry can have direct biological relevance. Transition metals such as Fe, Cu, Mn, and Co, are present in human tissues and serve as essential cofactors in numerous metalloenzymes responsible for redox regulation and cellular metabolism.^25^ The ability of 3-CP to chelate these metal ions suggests potential for interference with endogenous metalloproteins if the compound is formed in vivo, for example, during the vaping of nicotinamide-containing formulations. Such metal–ligand interactions could perturb metal homeostasis, inhibit enzyme function, or promote redox cycling through Fenton-like reactions that enhance ROS production.^25^ Collectively, these mechanisms provide a potential biochemical basis for the observed synergistic and pro-oxidative toxicity of nicotinamide and 3-CP coexposure, underscoring the importance of evaluating mixed chemical systems under realistic vaping conditions.

## Solvent and Flavorant Chemistry in E-Liquid Aerosol Formation

Propylene glycol (PG) and glycerol (GL) are the primary carrier solvents in e-liquids. During vaping, these solvents thermally decompose to form a range of degradation products, with the extent of breakdown influenced by device temperature (voltage or wattage), wicking efficiency and the PG:GL ratio.^26^ The degradation process often follows radical-mediated pathways, allowing interactions with O_2_,^27^ nicotine and its analogs that can yield distinct toxicant profiles. In addition to solvents, e-liquids contain numerous flavorant compounds with diverse functional groups, each capable of undergoing thermal degradation and contributing to a complex chemical mixture during aerosol formation. Importantly, while many flavorants are considered generally safe for ingestion (GRAS), their thermal degradation products and direct inhalation exposure raise significant toxicological concerns, as these compounds can reach the lungs without undergoing metabolic detoxification.

Currently there is little known about the interactions of 6-MN and NA with e-cigarette flavorants and additives. However, the lack of regulation of 6-MN and NA products can enable manufacturers to disregard flavor bans related to such products. It is thus important to understand the influence of nicotine on flavorant chemistry and toxicity as a foundation for determining the impact of 6-MN, NA and on flavors and toxicology. The chemistry of nicotine in the presence of flavorants is complex.^19^ Common flavoring agents such as *trans*-cinnamaldehyde, vanillin, benzaldehyde, and benzyl alcohol, combined with carrier solvents PG and GL, undergo thermal degradation during aerosolization^28^ (Figure 5). Previous studies have shown that PG and GL alone can form carbonyl compounds upon heating and aerosolization, and the inclusion of flavorants will amplify toxicant formation.^28^

**Figure 5:**
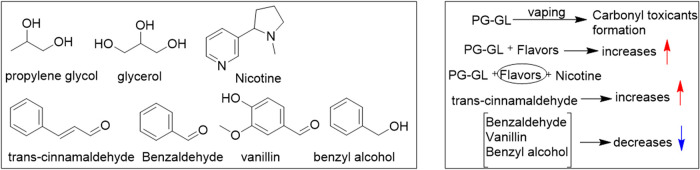
Selected relatively common chemical components of e-liquids addition to nicotine and varying effects on the formation of carbonyl toxicants.^28^.

Nicotine modulates the overall toxicant profile. For instance, *trans*-cinnamaldehyde acts as a radical scavenger and enhances the formation of carbonyl toxicants. In contrast, in the presence of vanillin, benzaldehyde, and/or benzyl alcohol, nicotine acts as a radical scavenger, reducing carbonyl emissions.^28–30^ However, this role of nicotine comes at the cost of its own degradation, suggesting a trade-off between toxicant suppression and alkaloid stability. This balance raises intriguing questions about whether structurally related analogs such as 6-MN and NA exhibit similar redox and radical scavenging behavior and how their interactions with various additives may influence both degradation pathways and toxicant profiles.

More recently, e-cigarette formulations have evolved to include synthetic coolants that are amide-containing compounds such as WS-23 and WS-3 (Figure 6), in addition to traditional menthol. This shared amide functionality introduces the potential for competitive radical or electron-transfer reactions as in amide atmospheric chemistry^31^ during thermal decomposition, as well as dehydration to cyano-derivatives as observed for 3CP formation from NA as described above and shown in Figure 3. The interactions between these amide compounds, flavorants, and carrier solvents under vaping conditions would influence radical formation, scavenging efficiency, and the resulting aerosol toxicant profiles in the presence of nicotine analogs. Understanding these interactions will be essential for evaluating the relative toxicological outcomes of emerging nicotine substitutes and their mixtures with solvents, flavorants, coolants and other additives.

**Figure 6:**
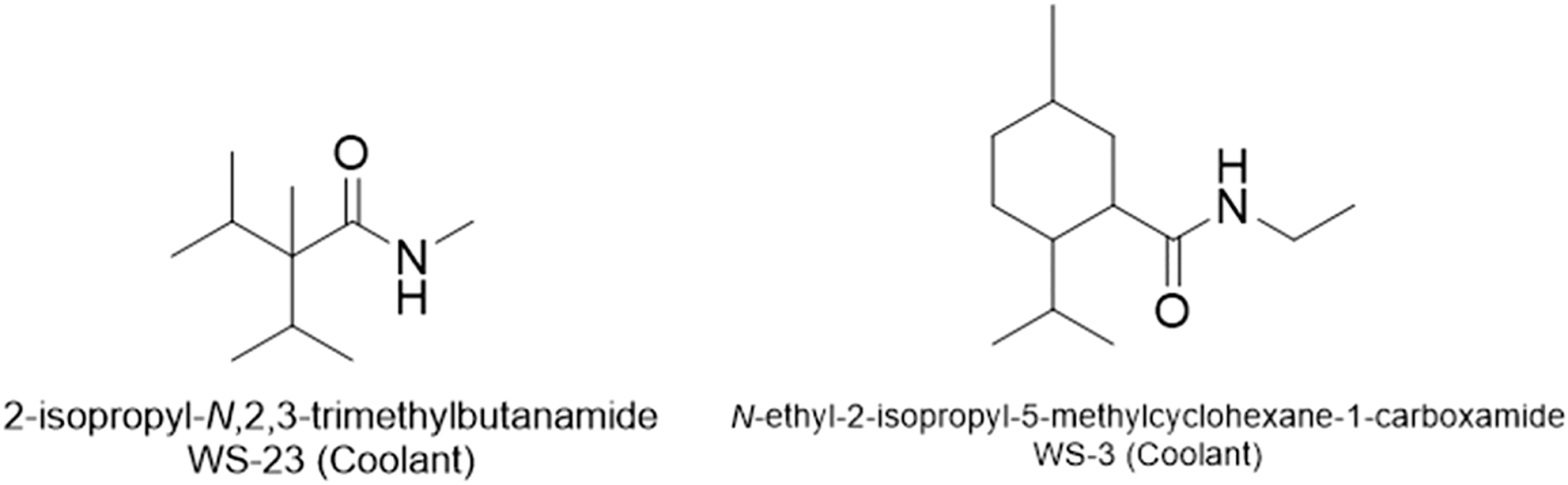
Chemical structures of coolants WS-23 and WS-3.

The presence of secondary amides such as WS-23 and WS-3 in e-liquids is thus of toxicological interest. A comprehensive understanding of the thermal stability and degradation chemistry of WS-type coolants is needed to help evaluate their safety profile.

## Influence of Nicotine Analogs and Their Salts on Flavorant Acetal Formation in E-Liquids

E-cigarette formulations incorporate not only free-base forms of nicotine analogs, such as 6-MN but also its organic acid salts, designed to reduce aerosol harshness and improve user tolerance. E-liquids contain propylene glycol (PG), glycerol (GL), and aldehyde-based flavorants, such as benzaldehyde and vanillin, which react with PG or GL catalyzed by organic acids (e.g., benzoic acid) to form acetal derivatives (Figure 7).^32^ These acetals possess distinct toxicological profiles, differing from their parent flavorant compounds.

**Figure 7:**
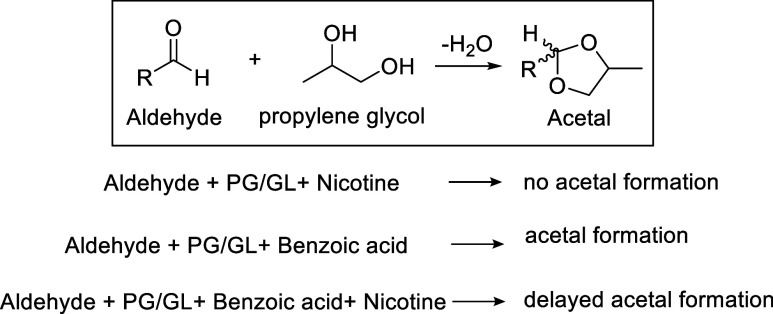
Effects on acetal formation in e-liquids in the presence of nicotine in free-base versus salt forms. Acetals form in e-liquids when aldehyde flavorants, such as benzaldehyde, vanillin, and trans-cinnamaldehyde.^33^

Conversely, in formulations containing free-base nicotine, nicotine can scavenge acid, thereby inhibiting or slowing the kinetics of acetal formation.^33^ Comparative investigations of nicotine salts and 6-MN salts will therefore be helpful to elucidate their influence on acetal chemistry within e-liquids and to better understand how these compositional differences impact toxicant generation under vaping conditions.

## Toxicology and Pharmacology of 6-MN and NA

Recent research indicates that 6-MN exhibits a pharmacological profile that may increase the risk of addiction and nicotine dependence due to its stronger potency and receptor binding affinity.^12,13^ Specifically, studies of methylated nicotine analogs concluded that while the most potent effects of receptor binding are varied by position of methylation, modification at the 6-position significantly alters the receptor binding and agonist efficacy to α4β2 nicotinic acetylcholine receptors^12^ (nAChR). α4β2 nAChR plays a significant role in mediating nicotine reward and dependence, while also contributing to cognition, mood, nociception, and reward.^34^ Hence, due to methylation of the 6-position for 6-MN, 6-MN may potentially be more potent and toxic compared to traditional nicotine,^13^ which could possibly translate to a stronger psychoactive and addictive potential.

Furthermore, as previously discussed, although the complete safety profiles of 6-MN are not entirely known and widely researched, in vitro studies of human bronchial epithelial cells suggest that 6-MN can increase ROS production, induce greater cytotoxicity, and reduce cell viability compared to conventional nicotine.^6^ This raises concerns of potential lung injury risks and oxidative stress when 6-MN is inhaled in abundant amounts. In other behavioral studies on rodents, exposure to 6-MN has been shown to elicit a dose-dependent behavioral change, altering activity and nociception, and demonstrating evidence of strong psychoactive effects.^35^ Interestingly, the self-administration of 6-MN at concentrations of 10 mg/mL is similar to the self-administration of nicotine, also suggesting similar addiction risks and reinforcing effects that are comparable to conventional nicotine.^35^

Nonetheless, despite the growing scientific attention to 6-MN’s pharmaceutical potency and toxicity risks, stricter oversight of 6-MN is still essential, as public health concerns regarding the carcinogenic and cytotoxic potential of 6-MN continue to rise since safety profiles of 6-MN-containing products are not fully known.

## 6-MN and Nixamide/Nixodine/Nicotinamide Commercial Availability

Commercial availability of 6-MN and other nicotine-analog products in the United States is currently dominated by the following primary distributors: Outlaw Dip, Kumi, Spree Bar, SBX, and Katchmi. These manufacturers offer products spanning oral nicotine pouches, disposable vapes/bars, and rechargeable pod systems, representing a range of delivery formats to replicate traditional nicotine products without tobacco-derived nicotine. Based on chemistry as described above, we further classified the emerging products into different categories with flavorings.

Outlaw Dip markets tobacco- and nicotine-free oral “fat cut” loose dip pouches containing 6-MN. These pouches are advertised as providing the sensory experience of traditional smokeless tobacco while eliminating tobacco and nicotine constituents. These products are available in a variety of flavors, including beverage-inspired, dessert, menthol, and traditional tobacco flavors, appealing to a broad user base. Each pouch is reported to contain 6-MN, as included in the product ingredient list alongside black tea leaves, salt, sodium carbonate, saccharin, propylene glycol, natural and artificial flavors, xylitol, and vitamin E.

Despite marketing claims that the active ingredient is NA in their “NIC-SAFE” label, the ingredient list does not reflect this. Instead, 6-MN is listed, a fundamentally different compound,^36^ raising questions about the accuracy and transparency of the product labeling.

Outlaw Dip also offers non-6-MN caffeine pouches, expanding its product line to cater to users seeking stimulation without nicotine and nicotine analogs.

Kumi focuses on disposable bars and rechargeable vape devices under the brand “Kumi Six” and offers products under the “Kumi Six Kurve” and “Kumi Six Scenic” lines. The Kumi Six disposables are prefilled with approximately 16 mL of e-liquid, delivering up to 10,000 puffs per device, with a 600 mAh rechargeable battery. The Kurve model expands the capacity to 28 mL and 35,000 puffs with a 900 mAh battery. The Kumi Six Scenic is the largest device in line, combining a 7 mL prefilled pod with a 15 mL refillable tank for a total e-liquid capacity of 22 mL, an 850 mAh battery, and an approximate puff count of 50,000. Kumi markets these devices as a nicotine-free substitute for conventional e-cigarettes, employing the term SFN (Substitute for Nicotine), or “NoNic6,” in place of nicotine and markets a concentration of 5% SFN by volume. Flavor offerings include mostly fruit and menthol profiles, mimicking conventional nicotine vaping experiences. All Kumi devices and nonrefillable, although rechargeable, offer greater convenience to refillable e-cigarettes.

Spree Bar markets 6-MN-containing products under the proprietary name “Metatine,” offered in 5% concentration Metatine for both disposable and rechargeable pod systems. The devices typically contain 12 mL of e-liquid and deliver up to 6,000 puffs per pod, with batteries aimed to last multiple refills. Spree Bar also contains products that are preconstructed, allowing users to use the product without the need to assemble the device. Flavor offerings are reminiscent of Kumi, replicating conventional e-cigarette products with fruit and menthol flavors.

SBX Vapes markets its product around Metatine and 6-MN-analog formulations, highlighting performance features in their products like adjustable airflow, dual-mesh coils, and high puff counts (20,000–25,000 puffs per device). SBX offers around 20 mL of prefilled e-liquid, a 1000 mAh rechargeable battery, and up to 25,000 puffs in its highest mode. Flavor portfolio includes 10–14 flavor varieties dominated by ice, fruit, and dessert profiles. Product labeling lists Metatine as the active ingredient but omits quantitative concentration data, instead emphasizing “clean vapor” and “nicotine-free satisfaction.”

Katchmi is a newer entrant marketing nicotine-free disposables (bars/vapes) under its “Nixodine” at around “50 strength”, which suggests a concentration of 5% or 50 mg/mL of “Nixodine”. Devices contain around 16 mL of e-liquid, which is equivalent to around 20,000 puffs, dual-mesh coils, and a 750 mAh rechargeable battery. Flavor offerings are similar to Kumi and SBX, emphasizing ice, frozen fruit, and energy drink profiles (Figure 8A–E).

**Figure 8:**
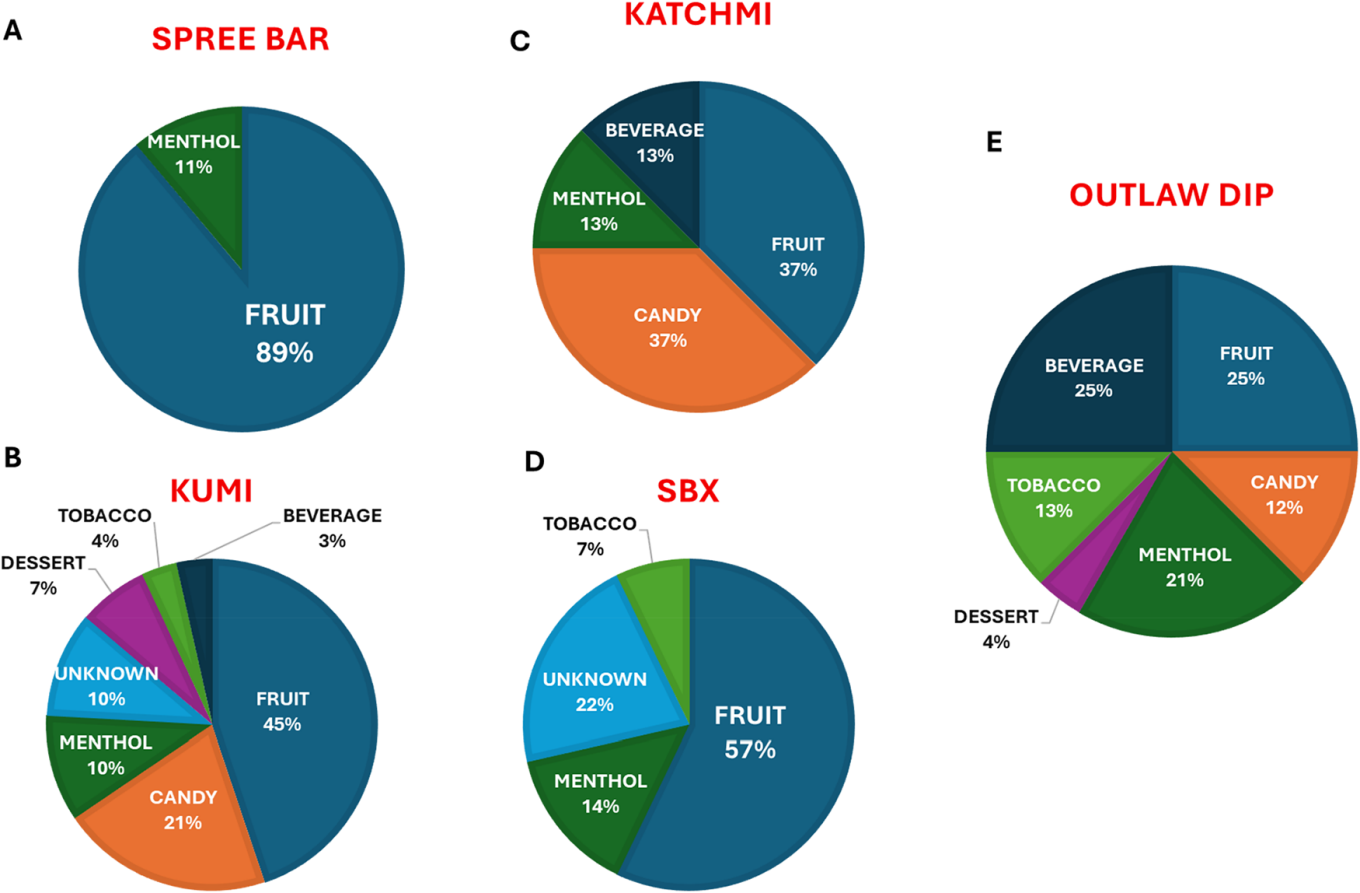
Series of pie-charts displaying the distribution of flavor categories for nicotine-analog containing products distributed by (A) Spree Bar, (B) Kumi-Six, (C) Katchmi, (D) SBX, and (E) Outlaw Dip.

Across these three distributors, 6-MN products vary in both delivery format and user experience (Table 2), but a common theme is the marketing of these synthetic nicotine analogs as “nicotine-free” alternatives.

**Table 2:** Table of Categorized Commercially Available Flavors from Products Containing Nicotine-Analogs

flavoring	brand	category
Strawberry Fanta	Kumi-Six	fruit
Blue Razz Ice	Kumi-Six	fruit-ice
Watermelon Ice	Kumi-Six	fruit-ice
Banana Ice	Kumi-Six	fruit-ice
Peach Ice	Kumi-Six	fruit-ice
Cool Mint	Kumi-Six	menthol
Sour Blue Gummy	Kumi-Six	candy
Tobacco	Kumi-Six	tobacco
Strawberry Watermelon	Kumi-Six	fruit
Triple Berry Ice	Kumi-Six	fruit-ice
Blue Razz	Kumi-Six	fruit
Menthol	Kumi-Six	menthol
Blue Kiwi Ice	Kumi-Six - Kurve	fruit-ice
B-Pop	Kumi-Six - Kurve	unknown/not listed
Pineapple Peach	Kumi-Six - Kurve	fruit
Baja Blue	Kumi-Six - Kurve	unknown/not listed
Sour Gush	Kumi-Six - Kurve	candy
Grape Rancher	Kumi-Six - Kurve	fruit
Strawberry Cake	Kumi-Six - Kurve	dessert
Sour Watermelon Gummy	Kumi-Six - Kurve	candy
Strawberry Watermelon	Kumi-Six - Scenic	fruit
Buns	Kumi-Six - Scenic	dessert
Grape Rancher	Kumi-Six - Scenic	fruit
Loopy Jammy Blue	Kumi-Six - Scenic	candy
Sour Rocket Pop	Kumi-Six - Scenic	candy
Cool Mint	Kumi-Six - Scenic	menthol
Baja Blue	Kumi-Six - Scenic	unknown/not listed
Vanilla Cola	Kumi-Six - Scenic	beverage
Sour Blue Gummy	Kumi-Six - Scenic	candy
Rainbow Fruit	Spree Bar	fruit
Blue Razz Ice	Spree Bar	fruit-ice
Strawberry Apple Melon	Spree Bar	fruit
Blood Orange Peach	Spree Bar	fruit
Watermelon Grapefruit	Spree Bar	fruit
Strawberry Mango	Spree Bar	fruit
Creamy Melon	Spree Bar	fruit
Sweet Spearmint	Spree Bar	menthol
Pineapple Coconut	Spree Bar	fruit
White Gummy Dream	Katchmi	candy
Acid Tears	Katchmi	candy
Sour Blue Cherry	Katchmi	candy
Miami Mint	Katchmi	menthol
Strazz Watermelon	Katchmi	fruit
Arizona Blast	Katchmi	beverage
Blue Razz Ice	Katchmi	fruit-ice
Watermelon Ice	Katchmi	fruit-ice
Blueberry Burst	SBX	fruit
Blue Razz Ice	SBX	fruit-ice
Clear	SBX	unknown/not listed
Confetti	SBX	unknown/not listed
Georgia Peach	SBX	fruit
Grape Ice	SBX	fruit-ice
Lemon Apple Ice	SBX	fruit-ice
Miami Mint	SBX	menthol
Mystic Ice	SBX	unknown-ice
Ripe Apple	SBX	fruit
Strawberry Kiwi	SBX	fruit
Sweet Spearmint	SBX	menthol
Summer Strawberry	SBX	fruit
Virginia Tobacco	SBX	tobacco
Wintergreen	Outlaw Dip - Fat Cut	menthol
Dark Wintergreen	Outlaw Dip - Fat Cut	menthol
Southern Sweet Tea	Outlaw Dip - Fat Cut	beverage
Killer Vaniller	Outlaw Dip - Fat Cut	candy
Ramblin’ Root Beer	Outlaw Dip - Fat Cut	beverage
Georgia Peach	Outlaw Dip - Fat Cut	fruit
Backwoods Blueberry	Outlaw Dip - Fat Cut	fruit
Wild Watermelon	Outlaw Dip - Fat Cut	fruit
Orange Creamsicle	Outlaw Dip - Fat Cut	fruit
Straight	Outlaw Dip - Fat Cut	tobacco
Gold	Outlaw Dip - Fat Cut	tobacco
Original	Outlaw Dip - Fat Cut	tobacco
Mint	Outlaw Dip - Fat Cut	menthol
Winterberry	Outlaw Dip - Pouches	fruit
Wintergreen	Outlaw Dip - Pouches	menthol
Apple Danish	Outlaw Dip - Pouches	dessert
Kraken Kola	Outlaw Dip - Pouches	beverage
Melon Ice	Outlaw Dip - Pouches	fruit
Red Deer	Outlaw Dip - Pouches	beverage
Sweden’s Fish	Outlaw Dip - Pouches	candy
Eagles Blood	Outlaw Dip - Pouches	beverage
Mead & Honey	Outlaw Dip - Pouches	beverage
Vanilla	Outlaw Dip - Pouches	candy
Mint	Outlaw Dip - Pouches	menthol

While most flavoring compounds discussed are generally regarded as safe (GRAS) for ingestion in food, their thermal degradation in subsequent inhalation in e-cigarettes introduces distinct and significant health risks. This section reviews the literature on the effects of flavorings and their additives in nicotine-containing e-liquids.

Aerosolization of nicotine analogs introduces chemical and toxicological effects such as elevated levels of ROS compared to nicotine and that flavored e-liquids can exert synergistic cytotoxic effects when combined with nicotine analog formulations.^6^ These findings suggest that flavorants-aerosol interactions may amplify oxidative stress and cytotoxicity. Additionally, recent GC-MS characterization of spearmint-flavored nicotine and 6-MN commercial products reveal further chemical complexity. GC-MS data of spearmint-flavored nicotine aerosols highlights substantial contributions from sensory additives menthol and carvone, and traditional flavor carriers like triacetin and methyl lactate. In contrast, spearmint-flavored 6-MN aerosols are dominated by a synthetic cooling agent, WS-23, and contain a broader array of monoterpenes and oxygenated terpenoids, including α-limonene, eucalyptol, β-myrcene, menthone, and menthol. The presence of both monoterpenes and oxygenated terpenoids highlights the chemical complexity of spearmint-flavored aerosolized 6-MN compared to aerosolized nicotine.^37^

While commercially available products from Outlaw Dip, Kumi-Six, and Spree Bar are all 6-MN formulations, there is currently no research specifically addressing the effects of flavorings on their constituent compounds in 6-MN e-liquids, highlighting a critical gap in the literature.

Fruit flavors dominate the flavor portfolios of both Kumi-Six and Spree Bar, with flavors, such as Strawberry Fanta, Blue Razz Ice, Peach Ice, Strawberry Mango, and Watermelon Grapefruit. Leigh et al. found that in ALI cultures of H292 human bronchial epithelial cells exposed to flavored e-liquids containing nicotine, Strawberry flavor was the most cytotoxic following neutral red assay and the release of interleukin-1 beta (IL-1ß), interleukin-10 (IL-10), C-Y-C motif chemokine ligand 1 (CYCL1), C-X-C motif chemokine ligand 2 (CXCL2), and C-X-C motif chemokine ligand 10 (CXCL10).^38^ Additionally, Leslie et al. found that in BEAS-2B cells, exposure to strawberry-flavored e-liquid resulted in reduced cell viability that was significantly greater than apple, cherry, and tobacco flavors.^39^ These effects are attributed to esters, aldehydes, and furans commonly used to replicate strawberry flavor,^40^ although the specific flavoring chemical eliciting the strong toxicity to strawberry-flavoring remains unclear. Thus, the frequent use of strawberry derivatives by Kumi-Six and Spree Bar likely places users at heightened risk of pulmonary toxicity.

Similar concerns extend to blueberry, raspberry, and mixed-berry flavors (e.g., Blue Razz, Backwoods Blueberry, Triple Berry Ice). Ganguly et al. determined that mixed fruit-berry blends in aerosol form produced transcription activation of inflammatory markers, such as IL-6, CXCL8, TNF-α, and IL-1ß, even in the absence of nicotine,^41^ suggesting that the flavoring chemicals themselves are major contributors. In other airway respiratory epithelial cells and relevant airway models, reduction in viability, increased ROS and oxidative stress, and mitochondrial dysfunction were observed.^42^ The chemicals most often found in berry-flavored e-liquids are methyl anthranilates^43^ and other berry esters known to trigger IL-6, TNF-α, and ROS pathways independent of nicotine,^44^ suggesting greater toxicity in Outlaw Dip, Spree Bar, and Kumi-Six products, potentiated specifically by berry-flavoring chemicals.

Menthol and mint products, including Cool Mint, Menthol, Sweet Spearmint, and Wintergreen (marketed across Kumi-Six, Spree Bar, and Outlaw), are highly characterized flavored e-liquid additives. These compounds are notable not only for their characteristic cooling sensation, but for their ability to suppress sensory irritation, thereby encouraging deeper inhalation and greater exposure to toxicants. Menthol consistently demonstrated disruption of epithelial integrity, induced cell death and cytotoxicity, and elevated ROS and enhanced secretion of IL-6 and IL-8.^45–47^

Candy and dessert-inspired flavors, such as Sour Blue Gummy, Strawberry Cake, Killer Vaniller, Sweden’s Fish, and Apple Danish, primarily include vanillin in vanilla-flavors, Furaneol in sweet-flavors, benzaldehyde in cherry/cola flavors, diketones in buttery/creamy flavors, and cinnamaldehyde in cinnamon-flavors.^48^ Benzaldehyde, diacetyl, and cinnamaldehyde are known respiratory health hazards according to OSHA.^49^ Flavor aldehydes, such as vanillin, ethyl vanillin, benzaldehyde, and cinnamaldehyde have been shown to be cytotoxic, suppress respiratory immune response, induce inflammation, and disrupt airway ciliary motility and mitochondrial function in various human bronchial epithelial models.^50^ Ortho-vanillin-treated BEAS-2B human epithelial cells induced greater IL-8 release compared to both controls and TNF-α.^51^ Furaneol, alongside ethyl maltol, maltol, ethyl vanillin, vanillin, and benzyl alcohol, formed the combinations that result in highly cytotoxic refill fluids for e-cigarettes, as tested in human BEAS-2B cells in MTT assays.^40^ In human bronchial epithelial cells, cinnamon flavored e-liquid exposures resulted in increased IL-8 secretion and decreased cell viability.^45^ Additionally, cinnamaldehyde has been reported to temporarily suppress epithelial cell ciliary motility through mitochondrial dysfunction.^52^ Similarly, vanillin and 2,5-dimethylpyrazine, commonly found in chocolate-flavors, has been observed to induce chronic CFTR signaling, which may negatively affect mucociliary clearance.^53^ Diacetyl’s occupational hazard and toxicity has been well-documented and provides the strongest causal evidence that inhalational exposure to diacetyl causes obliterative bronchiolitis.^54^

Beverage-inspired flavors including Southern Sweet Tea, Kraken Kola, and Red Deer, although less studied, share a similar toxicological profile to candy and dessert flavors because they use the same chemical building blocks, such as vanillin, benzaldehyde, cinnamaldehyde, and Furaneol. Outlaw Dip markets a particularly wide range of beverage flavors, including *Southern Sweet Tea, Ramblin’ Root Beer, Kraken Kola, Red Deer, Eagles Blood*, and *Mead& Honey*, while Kumi-Six Scenic offers *Vanilla Cola* and Spree Bar promote fruit-juice–style blends such as *Blood Orange Peach* and *Watermelon Grapefruit*. Many “tea/tea-like” e-liquids are constructed from vanillin, ethyl maltol, and fruity aldehydes rather than real tea extracts, whose toxicity has already been documented previously with candy and dessert flavors.^55^ Cola-flavored e-liquids have also been associated with biological effects, including significant mitochondrial dysfunction in airway epithelial cells, increased mitochondrial-induced apoptosis, and decreased phagocytosis and viability of macrophages, neutrophils, and natural killer (NK) cells.^44^

The lack of chemical compositional disclosure for many e-liquid flavors, particularly those in the Kumi-Six Kurve line, such as *Baja Blue* and *B-Pop*, provides little indication of their constituent flavoring additives. This lack of transparency poses a challenge for assessing toxicological risk, as the inhalation hazards of flavoring chemicals are highly compound-specific, highlighting a regulatory and public health concern in nondescriptive branding.

Coolants in ENDS and oral products encompass a wide range of compounds from naturally occurring menthol to synthetic cooling agents such as WS-23 and WS-3. These agents are added to nicotine-free e-cigarette products with flavor descriptors such as “Triple Berry Ice (Kumi-Six), “Melon Ice” (Outlaw Dip), “Blue Razz Ice” (Spree Bar), “Lemon Apple Ice” (SBX), and “Watermelon Ice” (Katchmi).^36^

Analysis of flavor offerings across 6-MN product lines indicates that mentholated and “ice” variants account for around 30% of available options. Kumi-Six and SBX’s products have 36% “iced”/menthol flavor variants, while Katchmi has 38% and Spree Bar has 22%. Outlaw Dip’s oral pouches have 21% mentholated flavors with no “iced” variants, underscoring the pervasive use of coolants even in noninhaled formats. The consistent inclusion of cooling agents across chemically distinct delivery systems suggests that the sensory function of these additives, by masking irritation and reinforcing flavor intensity, is central to the commercial appeal of nicotine-analog products.

Menthol and synthetic coolants act primarily through the transient receptor potential melastatin member 8 (TRPM8) ion channel, which mediate the sensation of cold and regulate airway irritation and nociception. Activation of TRPM8 elicits a cooling effect that can mask the harshness of inhaled aerosols, enable deeper inhalation and longer puff duration.^56^ Due to greater receptor potency and cytotoxicity observed in 6-MN, the sensory masking from coolants may consequently increase delivery of inhaled 6-MN.

Recent toxicological work by Yogeswaran et al. examined the interactions between tobacco-free nicotine formulations, chemically similar to 6-MN, and synthetic coolants, specifically WS-3 and WS-23. Their findings suggest that coolant-containing tobacco-free nicotine (TFN) aerosols generated lower levels of ROS in both acellular assays and human bronchial epithelial (BEAS-2B) cells, compared to TFN formulations without coolants.^56^ These results suggest that coolants may reduce oxidative stress by altering aerosol chemistry or reactivity.

However, these effects do not necessarily imply reduced harm. Studies by Muthumalage et al.^57^ and Yogeswaran et al.^56^ show that WS-3 and WS-23 can independently activate the NLRP3 inflammasome, alter macrophage migration, release inflammatory cytokines, and result in epithelial barrier dysfunction, even when ROS generation is unchanged or reduced.

Taken together, coolants may reduce chemical ROS but enhance biological stress via receptor-mediated or inflammatory pathways. No study to date, however, has examined coolant 6-MN mixtures directly, representing a key gap in understanding the toxicological implications of these emerging products.

## Emerging Nicotine Product Content and Labeling Disclosures

Emerging products, such as Spree Bar, Kumi-Six, Katchmi, SBX, and Outlaw Dip highlight the use of 6-MN or related analog as alternatives to tobacco-derived nicotine. These compounds are often branded under proprietary names, such as “Metatine,” “NoNic6,” “Nixodine,” or “6-MN/Nic-SAFE,” and are often described as “tobacco-free” or “synthetic nicotine alternatives.” Packaging and retail marketing frequently emphasize regulatory positioning, such as “PMTA-exempt” or “FDA-approved,” as well as perceived safety advantages. For example, Kumi-Six labels its Kurve line as “5% NoNic6,” Spree Bar claims “5% Metatine,” and Outlaw Dip presents its 6-MN pouches and e-liquids as smokeless or oral alternatives to traditional dip. Toxicological data, while still emerging, raise specific biological concerns. In vitro and transcriptomic studies comparing 6-MN with nicotine indicate that 6-MN can produce effects that are qualitatively like nicotine, in terms of activation of nicotinic receptors and downstream signaling.^58^ In some assays, 6-MN results in quantitatively greater receptor potency and perturbation of gene and protein expression.^6^ Exposures to aerosolized 6-MN-containing e-liquids in 3D human airway models have shown greater ROS generation and increased cytotoxicity compared to nicotine-containing aerosols.^6^

These strategies suggest innovation within the nicotine alternatives market, but they also raise questions about accuracy, consumer understanding, and regulatory oversight.

Independent chemical testing has revealed substantial discrepancies between labeled and measured 6-MN content. In a study of nine flavors of Spree Bar products claimed to have 5% 6-MN, researchers found that actual measure levels of 6-MN in all flavors were 87–88% lower than labeled.^36^ The same analysis identified additional constituents not disclosed on packaging, including artificial sweeteners not previously reported in US-marketed e-cigarettes, i.e neotame. These findings indicate that labeling may not provide a reliable guide to consumer exposure, complicating assessments of potential health effects for these products.

The implications of such discrepancies extend into the realm of marketing and consumer perception, which influence purchasing partners. Outlaw Dip, Spree Bar, and Kumi-Six all present themselves as alternatives to traditional nicotine products, but they employ strikingly different strategies to frame their appeal. Outlaw Dip positions its pouches and e-liquids primarily as replacements for smokeless tobacco, drawing heavily on themes of tradition and “rugged” authenticity. Its marketing particularly emphasizes regulatory legitimacy, frequently referencing FDA approval or compliance, and frames its products as tools for adult tobacco cessation. Implicit in this framing is a harm-reduction narrative, suggesting that based on the premise that a lower level of 6-MN is needed to achieve the same receptor activation as nicotine, nicotine analog products like 6-MN can be used as a nicotine cessation tool. However, this assumption does not account for the complex relationship between receptor activation, user behavior, and toxicological outcomes. Greater receptor potency does not necessarily translate to reduced usage. When 6-MN is used in lower amounts, the remaining volume of the e-liquid is comprised of other substances. This changes the chemical composition of both the liquid and the resulting aerosol, potentially increasing the concentration of other harmful components like heavy metals and flavorants. Emerging evidence indicates that commercially available nicotine analog products, often containing less 6-MN than disclosed, can still confer biological risk comparable to, or greater than, that of nicotine. In the absence of longitudinal inhalation data, harm-reduction claims predicated on reduced usage remain unsubstantiated. By contrast, Spree Bar, SBX, Katchmi, and Kumi-Six adopt branding strategies that emphasize flavor diversity, bright packaging, and “youthful” aesthetics. Their portfolios emphasize fruit, dessert, and beverage-inspired blends, which are widely recognized as highly appealing to younger demographics.^58^ Unlike Outlaw’s positioning as a harm-reduction or smokeless alternative, Spree Bar, SBX, Katchmi, and Kumi-Six frame their devices as lifestyle accessories, incorporating disposable (bars/vapes) hardware, sleek device designs, and advertising that aligns with the aesthetics of mainstream youth culture.

This divergence in marketing approaches underscores how different consumer groups may be targeted under the same “tobacco-free” and “synthetic nicotine” umbrella. In both cases, however, the combination of inconsistent labeling and limited toxicological data on 6-MN and other nicotine analog raises questions about potential misperceptions of safety and the adequacy of current regulatory oversight.

Regulatory and public-health authorities have noted the uncertainties surrounding nicotine analogs. The U.S. FDA and international agencies have flagged synthetic nicotine and nicotine products, viz 6-MN, as potentially more potent, and reflect concern that sales of such analogs could outpace evidence and oversight.

## Public Health Implications: Second-Hand and Third-Hand Exposures: Implications on Children and Adolescents

While risks to users of NA and 6-MN have been previously described in this review, an emerging concern involves the potential for secondary and tertiary exposures, particularly among children and nonusers. Secondary and tertiary exposure pathways associated with nicotine analog aerosols remain poorly characterized and are discussed here only as potential considerations. While indirect exposure has been documented for traditional e-cigarettes, comparable data for emerging nicotine analog products are lacking. However, given that 6-MN acts on similar receptors to nicotine and the established literature on nicotine and aerosolized residues,^59^ there is a strong basis for concern. Nicotine can deposit on surfaces, fabrics, and dust particles, creating persistent environmental contamination, also known as thirdhand smoke or aerosol exposure.^60^ These residues can be transferred through inhalation or skin contact, representing a low-level yet continuous exposure pathway. This implies that young children among the nonusers are especially vulnerable, due to higher hand-to-mouth behaviors, thinner skin, and greater respiratory rates per body mass.^61^

Second-hand exposure in pediatrics has been shown to result in metabolites of chemicals in e-cigarette liquids found in exposed children’s systems. These metabolites have been linked to disruption of dopamine levels and causing inflammation and oxidative stress, the latter of which is linked to numerous diseases, such as diabetes and cancer.^60^ Additionally, chronic exposure to low-level nicotine has been linked to altered neurodevelopmental outcomes, like deficits in attention, impulse control, and memory. These disruptions in neuronal signaling and synaptic plasticity are due to nicotine’s action on nAChRs during critical developmental windows.^60^ Because 6-MN appears to act on similar receptor subtypes, with sometimes greater potency, these risks could be amplified, especially if residues persist in household or school environments where children are present.

Moreover, labeling and branding strategies such as “tobacco-free,” “nicotine-free,” or “PMTA-exempt,” combined with “aesthetic packaging, can obscure the presence of potent psychoactive chemicals, leading caregivers and children to underestimate risk. Emerging evidence demonstrates that such descriptors reduce perceived harm, increased willingness to use nicotine-containing products, and lack of education on synthetic nicotine products, particularly among adolescents and young adults.^62^ Misleading claims of “nicotine-free” formulations may increase product accessibility to children, raising the risk for accidental ingestion. The brightly colored, candy-themed packaging common in Spree Bar and Kumi further heightens the potential for unintentional poisoning events. Data from U.S. poison control centers already show increasing pediatric nicotine exposures linked to flavored vapes, an alarming trend likely to extend to 6-MN products as they proliferate.^63–66^

From a public health perspective, these exposures pose a dual threat: (1) direct toxicity risks to children through inadvertent ingestion or dermal absorption, and (2) exposure from environmental persistence that extends beyond primary usage. Current labeling regulations,^67^ which often display federal exemptions e.g., “PMTA-exempt” or inconsistently classify synthetic nicotine analogs, hinder both surveillance and consumer education. Without clear disclosure requirements for nicotine analogs viz 6-MN, households may unknowingly introduce toxic agents into shared spaces with vulnerable nonusers.

## Regulation of Emerging Nicotine Analog Products

Current FDA regulation of nicotine products is largely grounded in solely tobacco-derived nicotine. As a result, emerging nicotine analogs like 6-MN, which are chemically distinct from nicotine but retain agonist activity at nAChRs, fall outside existing regulatory frameworks. This creates a regulatory loophole in which products capable of producing nicotine-like effects can be marketed without PMTA review by marketing their products as “nicotine-free” or as “not subject to FDA tobacco requirements.”^3^ Simultaneously, current research suggests these compounds produce similar receptor activity and possibly greater toxicological detriment than nicotine.^13,68^ To address this, we recommend that the FDA expand its product definitions to include any compound with agonist activity at nicotinic acetylcholine receptors intended for human consumption via either inhalation or oral use. Regulation should transition from regulating starting ingredients to regulating aerosol emissions. As this study demonstrates, ″safe″ precursors like nicotinamide can thermally degrade into hazardous pyridine-based toxicants (200 to 400 °C) that do not exist in the raw e-liquid.

Additionally, all products containing nicotine or nicotine analog should be required to provide comprehensive ingredient disclosure and quantitative labeling. Current labeling often omits critical information, such as active compound concentration, and independent testing has revealed significant discrepancies between labeled and actual contents.^36^ For vaping products, this should include active compound concentrations in mg/g, total mg per device or pod, and liquid volume (mL). For oral products such as dip or pouches, labels should specify total mg per can and mg per serving.

Flavor restrictions currently applied to nicotine-containing products should also be extended to include all nicotine analog, as nontobacco and nonmenthol flavors sold in nicotine analog products, are known to increase youth appeal as previously discussed.^5^

Finally, there is a need for rigorous toxicological studies to evaluate the health effects of nicotine analog, such as 6-MN and NA. These studies should include comparisons with nicotine and flavor additives, as well as ensuring these studies are conducted in physiologically relevant human models. Further investigation should also examine both the independent effects of 6-MN, NA and potential synergistic interactions with flavorings, with findings to be used to inform market authorization for such products, labeling requirements, and more comprehensive regulatory oversight.

## Conclusion

Nicotine analogs, such as 6-MN and NA have led to emerging challenges in the commercial nicotine and nicotine-like market. These compounds are marketed through diverse delivery systems, such as oral nicotine pouches, disposables (bars/vapes), and pod devices, and are often paired with fruit, candy, and beverage-inspired flavorings that obscure their chemical potency and appeal to consumers. Ambiguous labeling and proprietary branding further complicate the assessment of exposure and toxicity of these compounds. These products cause oral and lung toxicity due to generation of oxidants and cause inflammatory responses. This includes the activation of NF−κΒ, epithelial permeability, and lung remodeling due to ECM modifications. For example, methylated nicotine analogs exert the most potent effects and agonist efficacy to α4β2 nicotinic acetylcholine receptors (nAChR) for mediating nicotine addiction and toxicity. Expanding FDA definitions to include nicotine analogs, enforcing quantitative ingredient disclosure, and extending flavor regulations to synthetic products would improve regulatory oversight, enhance consumer transparency, and ensure commercial strategies do not outpace public health protections.

## Abbreviations

6-MN: 6-methyl nicotine
NA: nicotinamide
ENDS: electronic nicotine delivery system
e-cig: electronic cigarettes
CCR2: CC chemokine receptor 2
CCL2: CC chemokine ligand 2
CCR5: CC chemokine receptor 5
TLC: thin layer chromatography
PMTA: premarket tobacco product application
COPD: chronic obstructive pulmonary disease
FDA: Food and Drug Administration
TCA: Tobacco Control Act
GC/MS: gas chromatography and mass spectrometry
HRLC/MS/MS: high-resolution liquid chromatography coupled to tandem mass spectrometry
ROS: reactive oxygen species
nAChRs: α4β2 nicotinic acetylcholine receptors
3-CP: 3-cyanopyridine
HCN: hydrogen cyanide
NH3: ammonia
PG: propylene glycol
GL: glycerol
SFN: substitute for nicotine
ALI: air–liquid interface
OSHA: Occupational Safety and Health Administration
WS-23: 2 isopropyl N,2,3 trimethylbutanamide
WS-3: N-ethyl-2-isopropyl-5-methylcyclohexanecarboxamide
TRPM8: transient receptor potential melastatin member 8
IL-1ß: interleukin-1 beta
IL-10: interleukin-10
CYCL1: C-Y-C motif chemokine ligand 1
CXCL2: C-X-C motif chemokine ligand 2
CXCL10: C-X-C motif chemokine ligand 10
NK: natural killer
GRAS: generally recognized as safe
TFN: tobacco-free nicotine
ECM: extracellular matrix
